# Microbiome in a ground-based analog cabin of China Space Station during a 50-day human occupation

**DOI:** 10.1093/ismeco/ycae013

**Published:** 2024-01-24

**Authors:** Ying Zhang, Zhidong Li, Yuan Peng, Zimu Guo, Hong Wang, Tao Wei, Yasmeen Shakir, Guohua Jiang, Yulin Deng

**Affiliations:** School of Life Science, Beijing Institute of Technology, Beijing 100081, China; Office of International Business and Technology Application, Beijing Institute of Spacecraft System Engineering, Beijing 100094, China; School of Life Science, Beijing Institute of Technology, Beijing 100081, China; School of Life Science, Beijing Institute of Technology, Beijing 100081, China; School of Life Science, Beijing Institute of Technology, Beijing 100081, China; School of Life Science, Beijing Institute of Technology, Beijing 100081, China; Department of Biochemistry, Hazara University, Mansehra 21120, Pakistan; School of Medical Technology, Beijing Institute of Technology, Beijing 100081, China; School of Medical Technology, Beijing Institute of Technology, Beijing 100081, China

**Keywords:** China Space Station, microbiome, 16S rRNA gene amplicons ground-based analog manned cabin

## Abstract

Dead-corner areas in space station that untouched by the clean-up campaign often experience microorganisms outbreaks, but the microbiome of these areas has never been studied. In this study, the microbiome in a ground-based analog ``Tianhe'' core module of China Space Station was first investigated during a 50-day three-crew occupation. Dead-corner areas were receiving attention by adopting a new sampling method. Results indicate that the astronauts occupation did not affect the dominant bacteria community, but affected a small proportion. Due to the frequent activity of astronauts in the work and sleep areas, the biomarkers in these two areas are common human skin surface and gut microorganisms, respectively. For areas that astronaut rarely visits, the biomarkers in which are common environmental microbial groups. Fluorescence counting showed that 70.12–84.78% of bacteria were alive, with a quantity of 10^4^–10^5^ cells/100 cm^2^. With the occupation time extension, the number of microorganisms increased. At the same sampling time, there was no significant bioburden difference in various locations. The cultivable bioburden ranged from 10^1^ to 10^4^ colony forming unit (CFU)/100 cm^2^, which are the following eight genera *Penicillium*, *Microsphaeropsis*, *Stachybotrys*, *Humicola*, *Cladosporium*, *Bacillus*, *Planomicrobium,* and *Acinetobacter*. *Chryseomicrobium* genus may be a key focus for future microbial prevention and control work.

## Background

Planning for long-term manned on-orbit missions of future China Space Station (CSS) will require the construction of safely closed habitats to ensure the safety of astronauts and equipment. Mir space station (MIR) and the International Space Station (ISS) investigations have indicated that microorganisms are ubiquitous in these facilities and that their growth cannot be controlled by the available onboard equipment especially at the dead-corner areas in that untouched by the clean-up campaign [1, 2].

Human well-being is inseparably linked with its microbiome. As a typical human-crewed spacecraft, space station needs to have continuous astronauts living in the space station for a long time and performing on-orbit tasks. Therefore, the human microbiome will definitely affect the microbial ecological inside the cabin. Although human health is inseparable from the contribution of microorganisms, it has been reported that incidents of microbial contamination in MIR and ISS cabins have occurred. Dead-corner areas are particularly severe, such as hatch backboards, electronic circuit boards, cargo bags that astronauts do not frequently use, etc. These uncontrollable microorganisms will threaten the health of astronauts (such as urinary tract, upper respiratory tract, and subcutaneous skin infections) [3–6], they will also accelerate the corrosion of spacecraft equipment and seriously shorten the orbital service life of the space station [4, 6–10]. Therefore, reducing the harm of these microorganisms to human health and spacecraft is the focus of the space station’s microbial prevention and control work.

Due to the early launch of MIR and ISS, there is no comprehensive study on how the microbes inside them have become uncontrollable step by step, and how to prevent and control them to avoid microbial outbreaks. However, with the advancement of scientific methods and the promulgation, implementation, and promotion of planetary protection plans, the dynamics of the microbiome in and around a human being is the subject of research for the preparation of human long-term spaceflight settlement in remote locations [11]. Numerous suitable Earth-based model environments were used in the past to investigate microbiome [12]. These models are greatly differed in terms of task settings, setup and research design, etc. For instance, (i) the Concordia research station in Antarctica could accommodate 16–32 occupants, and its microbial monitoring experiment was subjected to 365 days [13]. (ii) The US inflated lunar/Mars analog habitat (ILMAH) with a total volume of 300 m^3^ and accommodated three occupants during a 30 day microbiological survey period [14]. (iii) The Mars500 with a total volume of 550 m^3^ and housed six occupants for 520 days [15]. (iv) The Hawaii Space Exploration Analog and Simulation IV (HI-SEAS IV) mission could accommodate six people in a dome (diameter: 11 m). The microbial community and the interactions of surface and skin microbiomes were assessed for 336 days. (v) Another example is the biological life-support testbed “Lunar Palace 1” (LP1) located in Beijing, China, which conducted many simulation experiments and tested microbime data for 105 and 365 days [16, 17]. It is worth noting that existing research on space environmental microbiome, whether in orbit or ground analog modules, sampling sites are all areas frequently touched by astronauts, but dead-corner areas were ignored.

In previous research, we analyzed air and surface samples from the structural test model of the core module of the CSS and the surrounding clean rooms during assembly [18]. However, it has never been reported what impact the astronauts will have on the CSS cabin after they are settled in the space station. It is still unknown how astronauts' work tasks and daily life will affect the microbiome in the CSS cabin. During the construction of CSS, several ground-based mock-up spacecraft and simulation habitats have been built mimicking most conditions prevalent during a spaceflight. Before the implementation of long-term on-orbit missions, a ground simulation crewed flight experiment program was conducted in a simulated core module of the CSS. The whole program was developed as a multistage. Its purpose was to acquire scientific and technical information. The first stage was a 50-day confinement study of a crew of three males in 2018. This project’s development is a very good microbiome research model, which can help us predict the microbiome changes when the CSS performs manned missions in orbit.

In this study, the microbiome of the simulation "Tianhe" core module of the CSS (hereafter referred to as the “analog cabin”), which was conducting a 50-day (medium- and long-term) manned simulation mission, was analyzed for the first time. Culture-based and molecular techniques were all used. Unlike previous studies, we have adopted a completely new sampling method, revealing for the first time the characteristics of microbial changes in the dead-corner area of the cabin. Our result opens a possibility for broadening the current surveillance practices to maintain the crew’s health and to promote advances in long space human habitation in the space station in the future.

## Materials and methods

### Sample location, collection, and processing

#### Sample location and collection

The samples were collected from the analog cabin. The size and general layout of the analog cabin were designed after the "Tianhe" core module of the CSS, including the hatch of the node cabin, the entire area of the living control cabin, and all the resource cabins. "Tianhe" core module is a cylindrical structure with a specific size of 16.6 m in length and a maximum diameter of 4.2 m. All simulation experiments were designed and organized by the China Astronaut Research and Training Center. The experiment was completed in many stages, and the samples for this study were collected from the first stage (a total of 50 days). The crew members involved in the first stage were three males.

**Figure 1:**
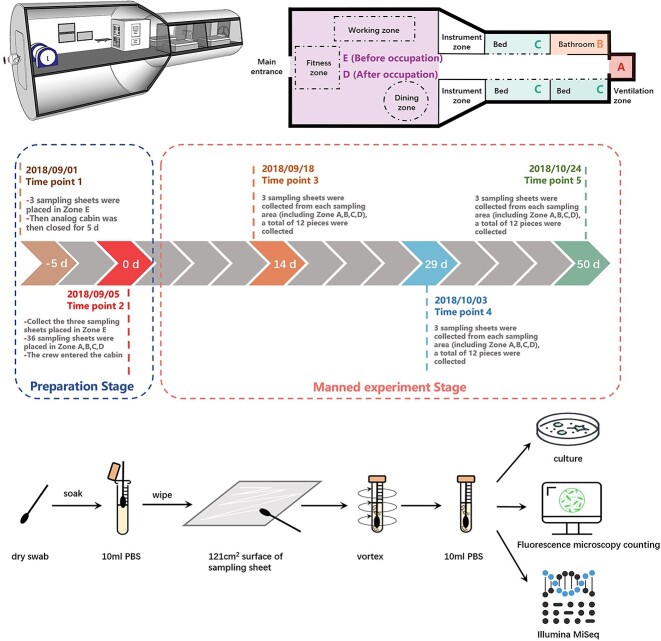
Overview of the analog cabin of CSS, experimental design and methods.

We used SketchUp (2019) software to draw a structural diagram of the analog cabin (Fig. 1 top left) showing the analog cabin’s approximate internal structure and the distribution of internal instruments from a three-dimensional perspective. Fig. 1 (top right) shows the four sampling areas of this microbiome experiment from a plane perspective, including the ventilation area (Zone A), the bathroom area (Zone B), the sleeping area (Zone C), and the working area (Zone D, Zone E). Zone A is located at the hatch of the node module, and its main function in the analog module is ventilation. Zone C and B together form the living control module area; Zone B is mainly the area for the crew to go to the toilet and clean-up personal hygiene; Zone C is located in the middle of the cabin, including three beds, and is the sleeping area for crews. The area representing the resource cabin is Zone D and E, these two areas are located on the other side of the cabin farthest from the ventilation area. The interior includes some instruments, fitness equipment, and a dining table. The main function is to provide crews with work, exercise, and dining places.

The manned simulation experiment lasted for 52 days in total. Three crews entered the analog cabin on 5 September 2018, and left on 26 October 2018. The sampling method in this study was to use a sampling sheet made of aluminum alloy with the same material as the surface of the analog cabin, which we called “Equivalent sampling sheet”, and the sheet size was 11 cm × 11 cm = 121 cm^2^. All sampling sheets need to be thoroughly sterilized before use. The specific method was: All the sheets were subjected to ultrasonic cleaning with double-distilled water at 80°C for 20 min, followed by a 97% ethyl alcohol rinse. These steps were repeated three times. Each sheet was packaged individually with tinfoil and placed in a big Petri dish before autoclaving at 121°C for 20 min followed by the aseptic removal of all the tinfoil. All sheets were dried with clean, dry nitrogen before placement back into the sterile Petri dishes. Before use, these sterilized sampling sheets were set as field blanks. When the set time points arrived, we aseptically took the sampling sheet out of the Petri dish, placed them at the dead-corners of typical areas in the analog cabin, and took them out at the specified time for subsequent microbiome analysis.

As shown in Fig. 1, the whole experimental process includes two experimental stages, with five time points (Fig. 1, Table 1). Disposable sterile gloves and sterile tweezers were used to place and collect the sampling sheets. The first experimental stage is the preparation stage, when the crews have not yet settled in. The first time task point occurred on 1 September 2018, 5 days before the crews officially entered the cabin. We placed three pieces of sampling sheet (E1, E2, E3) in the working area (E). The second experimental stage includes the second to fifth time task points. The second time point was 5 September 2018. We placed nine pieces of sampling sheet in the four sampling zones A, B, C, and D, respectively. For a total of 36 pieces. In addition, we took out the three sampling boards in the zone E placed at the first time point. On this day, after completing the operations of placing and retrieving samples, the crew officially entered the cabin, the day when the manned simulation experiment officially started (day 0). The third point of time was 18 September 2018, the 14th day after the start of the simulation experiment. We took out the second batch of sampling sheets. Specifically, three sampling sheets were taken from each of the four sampling zones A, B, C, and D, a total of 12 pieces of sampling sheets. The sampling sheet taken out were numbered A1, A2, A3; B1, B2, B3; C1, C4, C7; D1, D2, D3 (as shown in Fig. 1 and Table 1). The fourth point of time was 3 October 2018, the 29th day after the start of the simulation experiment. We took out the third batch of sampling sheets. Specifically, three sampling sheets were taken from each of the four sampling zones A, B, C, and D. A total of 12 pieces of sampling sheets were numbered A4, A5, A6; B4, B5, B6; C2, C5, C8; D4, D5, D6 (as shown in Fig. 1 and Table 1). The fifth point of time was 24 October 2018, the 50th day after the start of the experiment. Two days later, the crew left the cabin, and the first stage of the simulation experiment ended. We took out the last batch of sampling sheets. Specifically, three sampling sheets were taken from each of the four sampling zones A, B, C, and D, a total of 12 pieces were numbered A7, A8, A9; B7, B8, B9; C3, C6, C9; D7, D8, D9 (as shown in Fig. 1 and Table 1). It is worth mentioning that 10 days before the crew entered the cabin, routine sanitation work such as cleaning and wiping and microbial disinfection were carried out in the analog cabin. During the whole experiment, the crew members also had a strict cabin cleaning campaign schedule. However, since none of these cleaning and sterilization methods touched the equivalent sampling sheets we placed, these cleaning processes did not affect on our samples. As a negative control, during the simulation experiment, we placed three sterilized equivalent sampling sheets in a sterile petri dish and analyzed them together after the experiment.

**Table 1:** Abbreviation of the sampling area and sampling time.

**Sample serial number**	**Collection date**	**Sample location and (code)**	**Analytical method**
A_1_, A_2_, A_3_	14 d	Ventilation area (zone A)	Culture, fluorescence count
A_4_, A_5_, A_6_	29 d	Ventilation area (zone A)	Culture, fluorescence count
A_7_, A_8_, A_9_	50 d	Ventilation area (zone A)	Culture, fluorescence count, Miseq
B_1_, B_2_, B_3_	14 d	Bathroom (zone B)	Culture, fluorescence count
B_4_, B_5_, B_6_	29 d	Bathroom (zone B)	Culture, fluorescence count
B_7_, B_8_, B_9_	50 d	Bathroom (zone B)	Culture, fluorescence count, Miseq
C_1_, C_4_, C_7_	14 d	Sleeping area (zone C)	Culture, fluorescence count
C_2_, C_5_, C_8_	29 d	Sleeping area (zone C)	Culture, fluorescence count
C_3_, C_6_, C_9_	50 d	Sleeping area (zone C)	Culture, fluorescence count, Miseq
D_1_, D_2_, D_3_	14 d	Work area after occupation (zone D)	Culture, fluorescence count
D_4_, D_5_, D_6_	29 d	Work area after occupation (zone D)	Culture, fluorescence count
D_7_, D_8_, D_9_	50 d	Work area after occupation (zone D)	Culture, fluorescence count, Miseq
E1, E2, E3	–5 d	Work area before occupation (zone E)	Culture, fluorescence count, Miseq

### Sample pretreatment

Swabs are recommended for surface sampling according to the ECSS-Q-ST-70-55C standard [19] We swab-sampled the collected 39 sampling sheets with cotton swabs (Cat. No.: 806-WC; Puritan Medical Products, Guilford, ME). The pretreatment processes for samples are summarized in Fig. 1 (bottom). The cotton swabs were designed with a predetermined breaking point in shaft's center to ensure easy head separation after sampling. Two swabs were used for each sampling sheet. The head of each swab was premoistened with sterile phosphate-buffered saline (PBS) and rubbed over the entire area of the sampling sheet horizontally, vertically, and diagonally before snapping into 10 mL of sterile PBS. Specifically, swabs were first rubbed horizontally, then rotated (ca. 120°) and rubbed vertically, and similarly rotated again (ca. 120°) for diagonally rubbing. This rotation was performed to present a new area of the swab head for sampling. All the sampling tubes were then ultra-sonicated at 40 kHz for 2 min and thoroughly vortexed to detach microorganisms from the swab heads. The swab heads were then aseptically removed from suspension. Swabs that were premoistened only with PBS were used as a control, whereas swabs that were premoistened with PBS and exposed to the clean bench were used as handling controls.

After the sample pretreatment, we got 39 tubes of microbial suspension, and then we divided each tube of suspension into three sets for subsequent analysis (see Fig. 1 bottom). In the first part, we used a culture-based method to isolate and identify bacteria and fungi and count the number of culturable microorganisms. In the second part, we used a non-culture-based staining method, and then fluorescence microscopy was used to count the number of microorganisms and the proportion of living cells in all samples. In the third part, we used Illumina Miseq to detect the diversity of microbial community.

### Cultivable microbial examination

#### Cultivable bioburden measurements

Spread 2.7 ml of microbial suspension on Luria-Bertani agar medium (LB) (Cat. No.: 22700025, Invitrogen, USA), Reasoner's 2A agar medium (R2A) (Cat. No.: CM906, Oxoid, UK) and Potato dextrose agar medium (PDA) (Cat. No.: CM139, Oxoid, UK). The selection and composition of the medium refer to ECSS-Q-ST-70-55C standard [19] The culture temperature of LB and PDA medium was 37°C, and that of PDA medium was 28°C. The culture time was 7 days. During the incubation, colonies' presence were checked every 24 h. After the incubation, the number of microbial colonies on each Petri dish was counted. Media blanks were run for each medium and sampling event.

#### Culture collection and taxonomic analyses

Pure bacterial cultures from all agar plates were selected and the total DNA of each strain was extracted as described by Pospiech and Neumann [20]. The DNA from bacteria was further purified using a Bacteria Genomic DNA Extraction Kit (Cat. No.: DP302-02, Tiangen, China). The extracted DNA (~ 100 ng/μl, OD260/OD280 ratios = 1.80–1.96) was used as templates for subsequent analyses. The 16S rRNA genes of bacteria were amplified using the 27F and 1495R primers [21].

A total of ~5 mg (wet weight) of the purified fungal cell was sampled, frozen in liquid nitrogen, and then homogenized in a ball mill before extraction. The Qiagen DNeasy plant mini kit (Cat. No.: 69104, Qiagen) was used for genomic DNA extraction, following the manufacturer’s instructions. Purified DNA samples were used for subsequent molecular operations. DNA fragments of the ITS gene were amplified using the following ITS1 and ITS4 primers [22].

All amplicons were fully sequenced. Phylogenetic trees were constructed using the neighbor-joining method available in MEGA Version 5.05 and the Kimura 2-parameter model [23] after multiple data alignments by CLUSTALW [24]. The topology of the tree was evaluated using bootstrapping based on 1000 replicates. The accession numbers assigned to the partial 16S rRNA gene sequences were MK959034-MK959037, and MK968263. The accession numbers assigned to the partial ITS gene sequences were MK967559-MK967560, MK956938-MK956939, and MK956909-MK956910.

### Quantitation of total and viable microorganisms

Using a non-culture-based method; we counted the number of microorganisms and the proportion of viable cells in all samples. Based on the comparison of counting experimental methods in our previous study [25], the LIVE/DEAD® Bacterial Viability Kit (Baclight™) was used to stain the microorganism. A total of 1 mL suspension from each sample to mix with 1 mL of 2 × stock solution in LIVE/DEAD Bacterial Viability Kit L7012 (BacLight Invitrogen, Carlsbad, CA). Dyes were diluted to final concentrations of 6 and 30 mM for SYTO 9 and propidiumiodide staining, respectively. After 15 min of incubation in the dark, suspensions were filtered through a 0.2 mm Nuclepore black polycarbonate filter (Cat. No.: 110606, Whatman). Dilutions were made when the concentration of bacteria was too high to count under the microscope. The numbers of bacteria were estimated from counts of 10 microscopic fields (at 1000×) using three replicates for each sample. A Nikon microscope equipped with a halogen lamp, a 470–490 nm excitation filter, and a 520 nm barrier filter was used to count the number of green live cells and red dead cells, the sum of which is considered the total number of cells. A total of 1 mL sterile PBS solution and heat-killed (nonviable) isolates were used as a negative control. The number of bacteria per 100 cm^2^ of sample was calculated using the formula


$$ \mathrm{T}=\mathrm{N}\times \mathrm{A}/\mathrm{a}\div \mathrm{B}$$

where T is the number of bacteria/100 cm^2^, N is the average number of bacteria/field, A is the surface of filtration (mm^2^), a is the area of the microscopic field and B is the area of sampleing sheet (cm^2^).

### Molecular microbial diversity analysis

#### Illumina sequencing

Genomic DNA from 15 sample suspensions (Table 1) was extracted using an E.Z.N.A.™ Soil DNA Kit (Cat. No.: D5625–01, Omega Biotek, Doraville, GA, USA). All experimental procedures followed the manufacturers’ instructions except for the last step. Nucleic acid was eluted in 80 μL molecular grade water (pH 8.0) instead of Elution Buffer. The extracted DNA (~100 ng/μL, OD260/OD280 ratios =1.69–1.84) was used as templates for subsequent PCR. PCR was conducted using the 338f (5′-ACTCCTACGGGAGGCAGCAG-3′) and 806r (5′-GGACTACHVGGGTWTCTAAT-3′) primer set specific to the V3–V4 region of the 16S rRNAgene [26]. The samples were then analyzed using the Illumina high-throughput sequencing platform (Illumina Miseq PE300, USA).

#### Bioinformatic analysis and statistical analyses of illumina sequences

The raw data was divided into different samples according to the barcode sequence. Use Pear [27] (v0.9.6) software to filter and merge raw data. The sequences were removed from consideration if they contained ambiguous bases N, and the parts with low-quality scores (≤20) were cut out in the sequences. During merging, the minimum overlap setting was 10 bp, and the *P*-value setting was 0.0001. After merging, Vsearch [28](v2.7.1) software was used to remove sequences with length ˂230 bp and removed the chimeric sequence by uchime [29] method according to the Gold Database. Quantified sequences were processed with QIIME2 (2020.02 version). Demultiplexed reads were denoised with DADA2 [30] and an amplicon sequence variant (ASV) feature table was created. The BLAST [31] tool was used to classify all ASV representative sequences into different taxonomic groups against Silva138 Database [32], and an e-value threshold was set to 1e – 5. QIIME2 [28] was used calculate the richness and diversity indices based on the ASV information. Mothur was used to generate rarefaction curves. Heat map analysis of each sample's 20 most abundant genera and ASVs was conducted. Based on the results of taxonomic annotation and relative abundance, bar-plot diagram analysis was conducted. The β-Diversity distance matrix between samples was calculated using the Bray-Curtis algorithms and plotted PCoA. Analysis of Similarities (ANOSIM) of the Bray-Curtis distances was performed using the vegan package. Venn diagrams of the ASVs were created. All the above graphic were analyzed and created using R (v3.6.0) software. To detect the potential biomarkers, the linear discriminant analysis (LDA) and linear discriminant analysis effect size (LEfSe) method were used based on a normalized relative abundance matrix [33]. LEfSe analysis was applied based on the ANOVA test, Wilcoxon sum-rank test, and LDA. The threshold value of ANOVA and Wilcoxon sum-rank test was 0.05. The threshold of LDA was set as four in this study. We used Python (v2.7) software for LefSe [33]. The latest version of SourceTracker2 was used for source estimation of analog cabin samples. This analysis was performed using default settings with a rarefaction depth of 1000. Four hypothetical source environments were selected, including soil, human skin, human oral cavity, and human fecal, as they are considered potentially important sources of indoor microorganisms [34]. In short, the soil-derived taxa were from Zhang *et al*. [35], and the data from human-related sources were from Miller *et al*. [36], Wang *et al*. [37], and Hao *et al*. [38].

The sequences obtained by Illumina MiSeq sequencing were deposited in the National Center for Biotechnology Information Sequence Read Archive (SRA). All accession numbers are listed in Additional file 1: Supplementary Table S1.

## Results

All negative controls, including field blanks, media blanks, and handling blanks, showed no growth, and amplification of bacterial and fungal genes revealed no detection signal.

### Microbial burden

#### Culture-independent bioburden measurements and viability assessment

**Figure 2:**
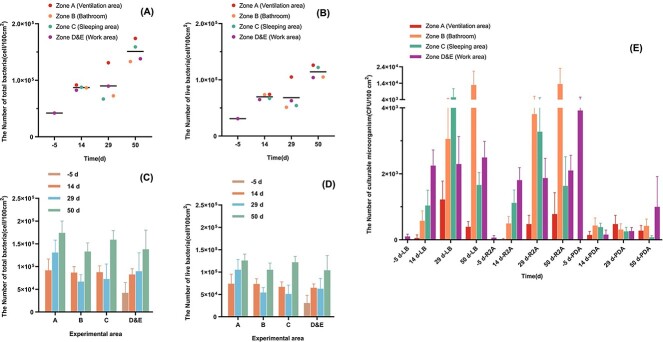
The number of microorganisms detected at each sampling point in the simulation cabin at different sampling times. (A) The number of total microorganisms detected by Baclight™. Samples are grouped by sampling time and colored by sampling location. (B) The number of live microorganisms detected by Baclight™. Samples are grouped by sampling time and colored by sampling location. (C) The number of total microorganisms detected by Baclight™. Samples are grouped by sampling location and colored by sampling time. (D) The number of live microorganisms detected by Baclight™. Samples are grouped by sampling location and colored by sampling time. (E) The number of microorganisms detected by culture-dependent method. The colored boxes represent the different sampling locations. The data of four different sampling times measured in the same culture medium are listed as a group.

Additional file 1: Supplementary Table S2 shows the results of counting the total number of microorganisms and the number of viable bacteria in the cabin using the Baclight™ staining kit. At the five sampling locations in the cabin, the number of living cells ranged from 3.09 × 10^4^–1.26 × 10^5^ cells/100 cm^2^, and the number of total cells ranged from 4.21 × 10^4^–1.74 × 10^5^ cells/100 cm^2^. It is worth noting that the microorganisms in each sample were basically alive; specifically, the proportion of living cells was 70.12%–84.78%. Fig. 2 (A–D) visually compares the change of bioburden in different sampling locations and sampling times in the cabin. Fig. 2A and B shows the changes in the number of microorganisms in the cabin as the simulation experiment time prolongs. The results showed that the average number of microorganisms in the cabin gradually increased with time, and the changing trend of the total number of bacteria was generally consistent with that of the number of viable bacteria. And at the same sampling point of time, there was no significant difference in the total and viable number of microorganisms in each sampling location (*P* > 0.05). Fig. 2C and D shows the changes in the number of microorganisms at each sampling location. The results showed that there was no significant difference between the number of microorganisms on the 14th day and the 29th day at all sampling locations (*P* > 0.05). In contrast, the number of microorganisms on the 50th day was significantly higher than that on the 14th day (*P* < 0.05). And the changing trend of the number of living bacteria and the number of total bacteria was always consistent.

#### Culture-dependent bioburden measurements

Fig. 2E and Additional file 1: Supplementary Table S2 shows the number of culturable microorganisms at different time points in different cabin locations. When the PDA medium was used, we found that the microorganisms did not change significantly with the extension of the experiment time after the crew settled in. When LB and R2A medium was used, the number of microorganisms in zone B and zone D showed an increasing trend with the prolongation. The highest values reached 1.35 × 10^4^ CFU/100 cm^2^ and 2.50 × 10^3^ CFU/100 cm^2^, respectively. The highest values of microorganisms in zone A and zone C appeared on the 29th day, with 1.22 × 10^3^ CFU/100 cm^2^ and 5.42 × 10^3^ CFU/100 cm^2^, respectively. It is worth mentioning that, before the crew entered the cabin (that is, the zone E), the number of culturable bacteria detected on the LB culture plate and the R2A culture plate was very low, 1.01 × 10^2^ CFU/100 cm^2^ and 64.4 CFU/100 cm^2^. However, the number of cultivable fungi detected on the PDA culture plate was the highest, which was 3.92 × 10^3^ CFU/100 cm^2^.

### Cultivable bacterial diversity

**Figure 3:**
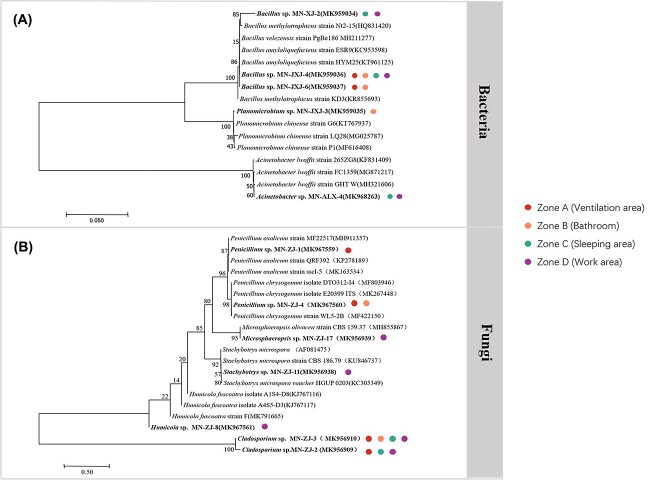
Phylogenetic tree of 16S rRNA genes showing the position of isolated (A) bacteria (B) fungi in the analog cabin. The tree was constructed using the neighbor-joining method and Kimura’s two-parameter model. Bootstrap values (obtained with 1000 sub-samples) >50% are indicated at the nodes. The scale bar indicates 1% nucleotide substitutions. The colored spots represent the location of these microorganisms isolated from the analog cabin.

The cultured microorganisms' 16S rRNA gene or ITS gene sequences were amplified. Then the obtained PCR products were sequenced. Then comparison of the obtained sequence with the sequence information in the GeneBank database was performed, and finally a phylogenetic tree was constructed. Fig. 3A shows the phylogenetic status of the five bacterial strains we identified, including *Bacillus* sp. 3 strains, *Planomicrobium* sp. 1 strain, and *Acinetobacter* sp. 1 strain. Fig. 3B shows the phylogenetic status of the seven fungi we identified, namely *Penicillium* sp. 2 strains, *Microsphaeropsis* sp. 1 strain, *Stachybotrys* sp. 1 strain, *Humicola* sp. 1 strain, and *Cladosporium* sp. 2 strains. At the same time, the source analysis of the culturable microorganisms, shows that the strains were widely distributed in the four sampling locations in the cabin.

### Bacteriome analysis

Amplicons of the V3–V4 region were subjected to next-generation sequencing (Illumina MiSeq PE300). A total of 2010 high quality reads of bacterial sequences of 400–440 bp in length were generated from 21 samples during this study. The sequences were clustered into 1973 ASVs.

The 21 samples were divided into six groups. Groups A, B, C, D, and E corresponded to the samples from zone A, B, C, D, and E on the 50th day of the simulation experiment. Each of these five groups of samples had three repeated tests. In addition to these five groups, we added six additional samples to be analyzed together, which were collected from the structural test model of “Tianhe” core modul. These six samples were collected in January 2016 [18]; at that time, the module was being assembled in Assembly Integration and Test (AIT) center. We refer to these six samples as “AIT cabin” samples for short and set their microbiome information as F group for analyzing the source and similarity.

#### Taxonomic profile of ISS microbial community

**Figure 4:**
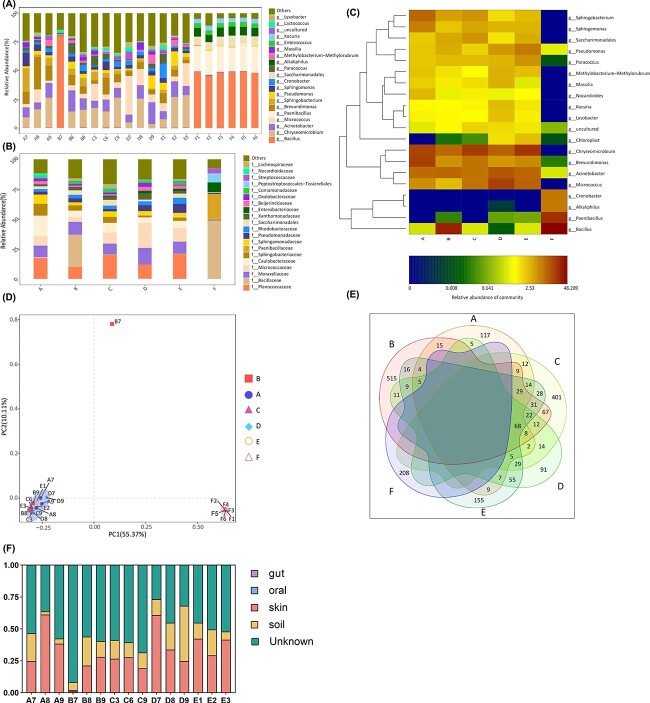
General characterization of the bacterial microbiome in analog cabin and the structural test model of "Tianhe" core modul. (A) Relative abundance of the major genus present in the bacterial microbial communities. (B) Relative abundance of the major family present in the bacterial microbial communities. (C) Heat map of the 20 most abundant genera in each group. The color intensity in each cell indicates the abundance of a genus in a group. The RDP classifier was used to assign sequences to different taxonomic levels at a 70% confidence level, and all the abundance was displayed as the percentage of the total sequences in each group. Groups represented by *X*-axis in the three figures above represent the A, B, C, D, E, and F groups. (D) Beta diversity estimates of the bacterial communities between different groups (ANOSIM test [with 999 permutations], significance threshold, *P* < 0.05). (E) Venn diagram of the numbers of ASVs shared. (F) The main sources of the microbiomes in the analog cabin as inferred by SourceTracker2.

The bacterial genus and family across all surface samples are shown in Fig. 4A and B. Fig. 4C shows each sample’s top 20 abundant genus in each sample in a heat map. The results showed that for the five groups of samples in the analog cabin, the total proportion of six genera of bacteria (including *Chryseomicrobium*, *Acinebotacter*, *Micrococcus*, *Paenibacillus*, *Brevundimonas*, *Bacillus*) accounted for >60% of the total number of bacteria. In addition, a small amount of bacteria belonging to seven genera *Sphingobacterium*, *Cronobacter*, *Pseudomonas*, *Sphingomonas*, *Alkalphilus*, *Saccharimonadales,* and *Paracoccus*. In contrast, the diversity of bacteria that account for a large amount in the AIT cabin (F group) was low, and the two genera of *Bacillus* and *Paenibacillus* are the main contamination. *Bacillus* genus, the major contamination in AIT cabin, also existed in the analog cabin. Still, its abundance was <1.21% in the other 14 samples, except for the B1 sample, which reached 79.74%. Bacteria of the genus *Chryseomicrobium* had a high abundance in the analog cabin, but this microorganism did not exist in the AIT cabin at all. It is worth noting that there were still a large number of uncultivable microorganisms in the analog cabin.

#### Beta diversity

Comparison between surface samples was visualized using principal coordinates analysis (PCoA) using Bray-Curtis distances (Fig. 4D). The results show that, except for the B1 sample, all the A, B, C, D, and E samples are gathered into a cluster. The results of ANOSIM using the Bray-Curtis distances agreed with the observed PCoA pattern (Additional file 1: Supplementary Table S3). Specifically, among groups A, B, C, D, and E, there was no significant difference between any two groups. (ANOSIM, pairwise test, permutations 999, *R*: from −0.148 to 0.482, *P*: from 0.101 to 0.786). The above results illustrated that no apparent dominant bacteria community differences were observed among locations in the analog cabin’s A, B, C, D, and E sampling locations. However, the six samples collected from the AIT cabin (F group) and the analog cabin samples (A–E group) show significant differences (ANOSIM, pairwise test, permutations 999, *R* = 1, *P* < 0.05). ANOSIM analysis of Bray-Curtis distances supported these observations, which found a significant difference between the F group and A, B, C, D, and E groups. The distance relationship within their groups was calculated for a multi-sample distance matrix with biological repeats (Additional file 2: Supplementary Fig. S1). The results showed that the intragroup differences in group B were relatively large. This result explains why the B1 sample is far from the B2 and B3 samples in the PCoA diagram. The main sources of the microbiomes in the analog cabin as inferred by SourceTracker2, were from soil and skin (Fig. 4F).

#### Alpha diversity

**Figure 5:**
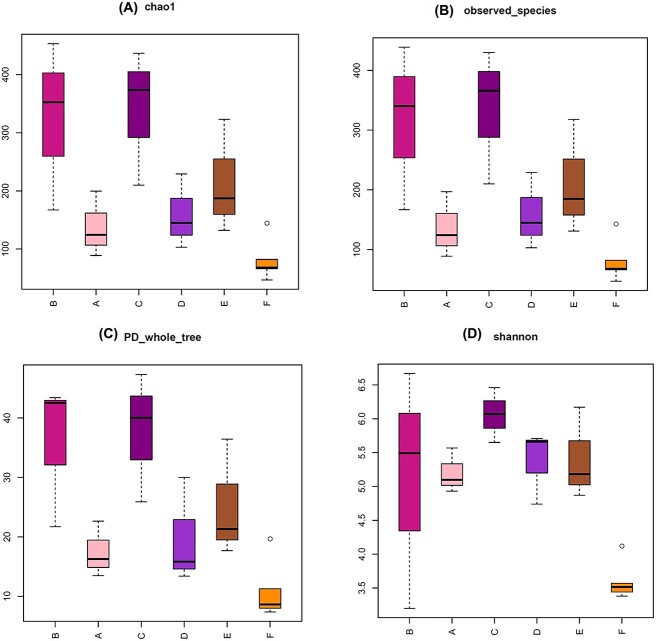
Alpha diversity metrics for A, B, C, D, E, and F groups. The chao 1 (A), observed species (B), PD whole tree (C), and Shannon index (D) are shown for each group.

The rarefaction curves, and Shannon-Wiener curves approached saturation at sequencing depth of 14 462 final tags. The shape of the species accumulation curve indicates that our sample was sufficient (Additional file 2: Supplementary Figs S2–S4). Four alpha diversity indices were used to measure the microbial diversity within each sample: chao 1 index (Fig. 5A) and observed species (Fig. 5B) indices reflect the ASV abundance (species richness) in samples, PD whole tree (Fig. 5C) reflect the phylogenetic diversity and Shannon index (Fig. 5D) reflect the species diversity. All Boxplots show that the C group had more abundance and diversity than others. The number of ASVs overlapping between group C and A, B, C, D, and E was also relatively large (Fig. 4E Venn diagram). F group had the lowest abundance and diversity.

### LEfSe analysis

**Figure 6:**
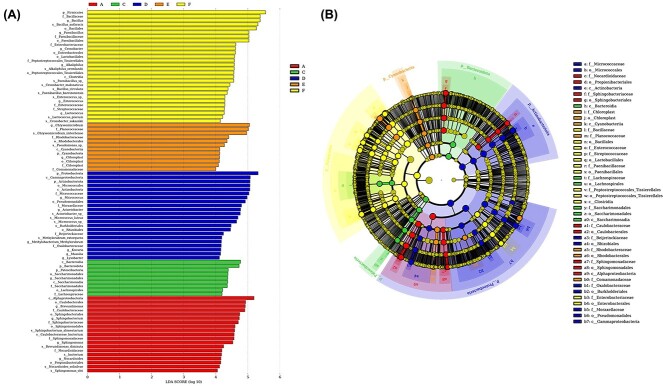
LefSe analysis. LDA score is >4. LEfSe analysis used the alpha value of 0.05, and the comparisons were performed in pairwise using Wilcoxon sum-rank test. (A) LEfSe identified the classified bacterial taxa differently among 6 groups. (B) The cladogram depicts the structure of the microbiota.

To determine the classified bacterial taxa with significant abundance differences between the six groups of samples, we performed biomarker analysis using the LDA effect size (LEfSe) method. Fig. 6A depicts the greatest differences in multiple taxa levels among the six groups. The AIT cabin was characterized by a high abundance of *Bacillus*, *Paenibacillus*, *Alkaliphilus*, *Enterococcus*. The work area before occupation samples were characterized by high abundance of *Chryseomicrobium* and *Pseudomonas*. Indicative microbial signatures were identified for the work area after occupation (*Micrococcus*, *Acinetobacter*, *Kocuria*, *Methylobacterium*, *Lysobacter*, *Massilia*), sleeping area (Bacteroidia, Lachnospiraceae), ventilation area (*Brevundimonas*, *Sphingobacterium*, *Sphingomonas*, *Nocadioides*). As shown in Fig. 6B, the cladogram depicts the structure of the microbiota.

### Co-occurrence of microbial taxa

**Figure 7:**
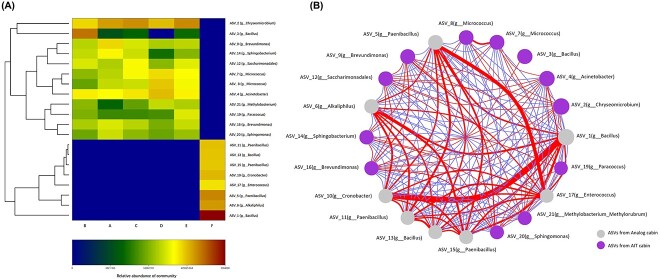
(A) Heat map of each group’s top 20 most abundant ASVs in each group. The color intensity in each cell indicates the abundance of a genus. (B) Correlation network analysis between the top 20 ASVs (*P* < 0.05, Spearman’s coefficient >0.4). Each node represents taxa affiliated at the genus level (based on 16S rRNA), and the size of each node is proportional to the relative abundance of the genus.

To understand the interaction relationship between the high-abundance microbial strains in the sample, we examined the correlations between the top 20 ASVs. The top 20 ASVs and their abundance in each group are shown in the heat map in Fig. 7A. The eight ASVs with higher abundance in AIT cabin (F group) are g_*Bacillus*(ASV_1), g_*Paenibacillus*(ASV_5), g_*Alkaliphilus*(ASV_6), g_Cronobacter(ASV_10), g_*Paenibacillus*(ASV_11), g_*Paenibacillus*(ASV_15), g_*Bacillus*(ASV_13), and g_*Enterococcus*(ASV_17) from high to low. These eight ASVs were absent in the analog cabin (A, B, C, D, E group). The 12 ASVs with high abandance in the analog cabin are g_*Chyseomicrobium*(ASV_2), g_*Acinetobacter*(ASV_4), g_*Micrococcus*(ASV_8), g_*Micrococcus*(ASV_7), g_*Bacillus*(ASV_3), g_*Brevundimonas*(ASV_9), g_*Sphingobacterium*(ASV_14), g_Saccharimonadales(ASV_12), g_Methylobacterium(ASV_21), g_*Paracoccus*(ASV_19), g_*Brevundimonas*(ASV_16), g_*Sphingomonas*(ASV_20). Correlation between the top 20 ASVs was shown in Fig. 7B, The red lines between the nodes indicate positive connections among the genera. The blue lines between the nodes indicate negative connections among the genera. It can be seen from the network diagram (Fig. 7B) that the seven high-abundance ASVs (gray nods) in the AIT cabin are negatively correlated with the high-abundance ASVs in analog cabins (purple nods). It is worth noting that the ASV_2 with the highest abundance in the analog cabin was *Chryseomicrobium*, which also has a relatively high positive correlation with other microorganisms, it has the most positive correlations with other microorganisms, and the correlation coefficients of these positive correlations are all relatively high (Fig. 7B and Additional file 1: Supplementary Tables S4 and S5).

## Discussion

Several previous studies have reported the microbial contamination data on orbiting and ISS and MIR some manned simulation experiment cabins on the ground [9, 15, 39-44]. It is worth mentioning that the microbial sampling method used in this study is different from most existing experiments. Previous studies used cotton swabs or wet wipes to wipe the surface or the floor to retrieve samples directly. Then they brought them back to the laboratory for analysis or used contact plates to culture them in orbit directly. There is a cleaning campaign schedule for both the orbital cabin and the ground analog cabin, and the microbial disinfection operation will be carried out in the cabin, including the microbial sampling area. The disinfecting operation will change the microbiome of the sampling site. However, the actual situation in the orbiting space station is that the dead-corners that astronauts do not pay attention to are more likely to breed microorganisms, and these dead-corners are often missed in daily cleaning campaigns, or cannot be cleaned at all due to the design of some narrow structures in the cabin. In this study, the Equivalent sampling sheet was placed in the dead-corners of the cabin. The cleaning campaign did not touch the equivalent sampling sheet during the whole manned simulation experiment. Therefore, our results can reflect the microbial contamination in dead areas that cannot be cleaned.

In the early days of ISS and MIR construction, culture-based methods were used to detect the degree of microbial contamination in the cabin [2, 6]. During the first 15 years of MIR's orbital operation, the number of culturable bacteria and fungi in the cabin was 10^4^–10^8^ CFU/100 cm^2^. Several studies have been carried out to measure the microbiological cleanliness of the ISS environment using cultivation-based approaches since the inception of this closed system [45]. In a recent paper, it was reported that the concentration of cultivable bacteria and fungi on the inner wall surface of the ISS cabin that has been in orbit for 17 years is 10^2^–10^7^ CFU/100 cm^2^ [46]. For this 50-day simulation, we detected 10^1^–10^4^ CFU/100 cm^2^ of culturable bacteria and fungi. Compared with the space station that has been in orbit for ˃10 years, the ground-based simulation module that has only been used for 50 days is obviously much cleaner. During the MARS500 and ILMAH simulation experiment, the degree of culturable microbial contamination of these ground-based simulation cabins was close to that of our study.

In this study, we used fluorescence counting as a culture-independent method to detect the number of microorganisms in the sample. The choice of this experimental method was based on previous method validation experiments [25]. The results showed that the microbial live cell concentration in the analog cabin was 10^4^–10^5^ cells/100 cm^2^. The average level of microbial contamination in the ISS cabin that has been in orbit for 17 years was 7.1 × 10^9^ 16S rRNA gene copy number /100 cm^2^ [46]. The microbial contamination level in the cabin was 10^2^–10^4^ 16S rRNA gene copy number/100 cm^2^ on the 30th day of the ILMAH simulation experiment. These research data were detected by the qPCR method. Considering that some microorganisms have multiple copies of the 16S rRNA gene (1–15 per genome) [47], The results of our experiment this time are more similar to the contamination levels of MARS500 and ILMAH. The above results prove that the level of microbial contamination in the ground-based simulation experiment is lower than that in orbit, possibly due to the short experiment time. It is worth noting that in most of the studies of previous researchers, whether it is the data in the orbital cabin or the data in the ground-based analog cabin, microbial contamination is all continuously fluctuating. However, since our samples were not cleaned, the microbial contamination we detected continued to rise. At the same time, most of the microorganisms detected in the analog cabin were alive, the same as the results of many previous reports on microbial contamination of the space environment [42, 46]. The above results show that all areas in the space station do need to disinfect microorganisms.

Regarding the types of culturable microorganisms, the *Penicillium,* and *Bacillus* we isolated and identified are common culturable microorganisms in the space station environment. The genus of *Microsphaeropsis* fungal strain we isolated has the highest similarity to *Microsphaeropsis olivacea*, which is a common microorganism in the environment that may infect human skin [48]. *Stachybotrys* and *Humicola* are also common fungi in the environment, and no pathogenicity has been reported. The genus of *Cladosporium* is a genus of fungi including some of the most common indoor and outdoor molds. Some species are endophytes [49] or plant pathogens, while others parasitize fungi. *Planomicrobium chinense* is a Gram-positive, aerobic, and motile bacterium from the genus of *Planomicrobium*, which has been isolated from sediments from the coast of the Eastern China Sea in China. *Acinetobacter lwoffii* is considered normal skin flora, and it can cause infections in human hosts, particularly catheter-associated infections in immunocompromised patients [50, 51]. This strain was also reported to have the ability to survive dry conditions, low pH, and a wide range of temperatures. It is also resistant to many disinfectants, irradiation, and desiccation [52]. These microorganisms will likely appear in the orbiting CSS cabin in the future, so exploring some effective ways to kill these pathogenic bacteria in subsequent experiments is necessary.

When we compared the microbiome profiles of each dead-corner in the analog cabin, the PCoA analysis was used to compare the beta diversity based on Bray-Curtis distances. We found that beta diversity was not significantly different among A, B, C, D, and E groups. Since Bray-Curtis distance based beta diversity will be influenced primarily by the most abundant species, we know that the dominant bacteria community was shared across each and every area in analog cabin. Since there was no significant difference in the microbial communities between the crew's pre-occupation cabin and the crew's 50 day post-occupation cabin, proving that humans will not affect the dominant bacterial community of the dead-corner in the cabin during medium to long-term manned missions. This research result is not entirely consistent with the results of ILMAH, Mars500, and LP1(365d) [15, 16, 18]. The research results of ILMAH indicate that human occupation can significantly affect the microbiome in the cabin in just 13–30 days. The Mars500 microbiome was found to be influenced by a plethora of different factors, including the surface material, the location within the facility and/or the function. The research results of LP1(365d) indicate that plants are the most crucial source of the surface fungal microbiome. Our research conclusion differs greatly from the previous results of other simulation modules, and we speculate that the main reason for this difference may be due to the significant differences in microbial sampling methods compared to previous studies. Previous studies have all directly collected surface samples from the cabin, and astronauts touch these sampling areas in their normal work and life. In this study, the equivalent sampling sheets placed in the cabin in advance were not frequently touched by crews in their daily work and life. In summary, our experimental results may be more representative of the microbial community changes on the surface of some cabin walls that astronauts do not frequently touch or clean. In addition, there are no plants in the analog cabin of this study, and the materials used for all microbial sampling sheets are the same, which avoids the impact of plant and surface material composition on the microbiome. At the same time, since human’s dispersal of microorganisms depends on their activities and time spent in the closed habitats, the crew's activities and tasks during the execution of each project and the residence time may also affect the distribution of microorganisms.

However, when we analyzed the alpha diversity results, we found that the rank abundance curves differ among different groups (Additional file 2: Supplementary Fig. S5). When we limited our focus to analyzing the differences in microbial communities in various locations of the analog cabin in this experiment, we found that the results of alpha diversity showed that the number of microbial ASVs in the sleeping area (zone C) was significantly higher than that in ventilation area and work area (zone A, D, and E). From physiological and specific diversity perspectives, these two values in Group C are also significantly higher than those in Group A and D. There are fewer rare species in A and D groups. This Illustrates that there were differences in the microbial community of each location within the analog cabin, even though the dominant community members are likely shared. This difference is mainly reflected in some lower abundant organizations. To identify which microorganisms in each location have significant differences, we further conducted LEfSe analysis. We found that c_ Bacteroida, f_ Lachnospiraceae are some high-abundance bacteria in the sleeping area but not in other locations. It is worth noting that these two bacteria are all commonly found in the human gut microbiota [53-55]. The biomarkers of the work area after occupation were *Micrococcus*, *Acinetobacter*, *Kocuria*, *Methylobacterium*, *Lysobacter*, *Massilia*. Most of these microorganisms exist on human skin and the oral cavity [56]. The way the crew works in the workspace happens to be primarily through touch. Biomarkers in work areas without occupation and ventilated areas are bacteria ubiquitous in the environment [57-59]. In summary, there was a clear correlation between biomarkers in various locations and human activities in corresponding locations.

Another interesting issue is that Zone B is the location for astronauts to wash and use the bathroom, which was originally considered the most susceptible area for microbial growth in the space station environment and should be cleaned up frequently. The entire cabin was cleaned once a month. However, there was no significant difference in the number of microorganisms between Zone B and other areas, and there was also no biomarker in Zone B.

The difference in microbial community between AIT cabin and this analysis cabin was significant. Significant differences existed from various perspectives, such as Alpha diversity and Beta diversity analysis. The number of overlapping ASVs was also zero (Fig. 4E). In the study published in 2022 by Yang *et al*., a comparative analysis of microbial communities in different spacecraft environments was conducted, and the results showed significant differences in microbial communities in spacecraft environments such as AIT, ISS, ILMAH, and LP1 [17], which is in line with our conclusion. It is speculated that the environment in which the spacecraft is located will directly affect the microbial community inside the spacecraft.

The results of the taxonomic analysis indicate that the high abundance of bacteria identified in the analog cabin has a high similarity at the family level with those identified in various spacecraft environments, including in orbit ISS, ILMAH, and many simulated Mars flight spacecraft [15, 40, 46, 60]. Specifically, almost of the top 30 high-abundance families in this analog cabin can be found in the ISS and ILMAH environments. However, there are significant differences in the proportion of these microorganisms.

As is well known, there are interactions between microorganisms living in the same environment. Fig. 7A revealed the relationship between the top 12 ASVs in the analog cabin. It can be seen that many high-abundance bacteria in the environment that have a positive correlation with two strains belonging to *Chryseomycobium* genera. Exploring co-occurrence patterns between microorganisms in ananlog cabin can help us identify potential biotic interactions, habitat affinities, or shared physiologies that could guide more focused studies or experimental settings [61]. For example, if we can find an effective way to kill microorganisms belonging to the *Chryseomycobium* genera, microorganisms with strict reciprocal symbiosis realtionships with them may disappear on their own. For another example, when isolating microorganisms from the space station environment, we can consider co-culturing these two microorganisms with other previously uncultivable microorganisms, which may result in more pure cultivation.

Since the experimental environment of this study is the closest to the manned experimental environment of the CSS in orbit among all reports so far, the research results obtained in this study have theoretical guidance significance for the microbial prevention and control work of the CSS's medium- to long-term manned space missions. However, due to the different experimental conditions between the analog cabin and the in-orbit CSS, for example, the analog cabin had no radiation and microgravity environment. It was not completely close as the in-orbit space station, the layout in the cabin was not 100% consistent with the in-orbit space station, and the crew of astronauts participating in the mission was also not consistent; these factors would cause the experimental data not fully to represent the in-orbit state. In future research, we will further analyze the CSS in orbit. Meanwhile, samples from multiple microbial sources should be collected more comprehensively, so that the sources of microorganisms can be analyzed more comprehensively and accurately.

## Conclusion

Our study participated in a 50 days space manned simulation experiment conducted in the ground-based simulation module of the CSS "Tianhe" Core Module. We focus on the microbiome data in the dead-corner area of the cabin through a new sampling method. The results reveal the following experimental conclusions: (i) The astronauts' 50-day occupation will not affect the dominant bacteria community in the dead-corner of the cabin, but will affect a small proportion of microbial communities. (ii) There was a clear correlation between biomarkers in various locations and human activities in corresponding locations. (iii) 70.12–84.78% of bacteria in the analog cabin were alive, with a quantity range of 10^4^–10^5^ cells/100 cm^2^. (iv) With the extension of astronaut occupation time, the number of microorganisms in dead core areas increased. (v) There was no significant difference in the number of microorganisms in various locations. (vi) The cultivable bacterial and fungal bioburden ranged from 10^1^ to 10^4^ CFU/100 cm^2^, which are the following 8 genera *Penicillium*, *Microsphaeropsis*, *Stachybotrys*, *Humicola*, *Cladosporium*, *Bacillus*, *Planomicrobium,* and *Acinetobacter*. (vii) There was a significant difference in the microbiome between the analog cabin and the AIT cabin.

## Supplementary Material

additional_file_1_ycae013

additional_file_2_ycae013

## Data Availability

The raw sequencing data has been deposited in NCBI SRA under accession number SRR11856650, SRR11856723, SRR11856722, SRR11856721, SRR11856720, SRR11856719, SRR11856718, SRR11856717, SRR11856716, SRR11858530, SRR11858539, SRR11858538, SRR11858537, SRR11858536, SRR11858535, SRR5469191, SRR5469170, SRR5469097, SRR5469171, SRR5469169, SRR5469154.
