# Supplementary material for: Microbiome in a ground-based analog cabin of China Space Station during a 50-day human occupation
Source: ISME Commun. 2024 Jan 24;4(1):ycae013. doi: 10.1093/ismeco/ycae013 (PMC10942772; doi:10.1093/ismeco/ycae013)
Supplement: additional_file_1_ycae013 [file additional_file_1_ycae013.docx]

**ADDITIONAL FILE 1**

Supplementary Table S1 Illumina Miseq raw data accession number, ASVs data and average ASVs data table within the group corresponding to all samples

| Sample serial | Accession # | ASVs | Mean OTUs within the group |
| --- | --- | --- | --- |
| E_1_ | SRR11856650 | 318 | 211 |
| E_2_ | SRR11856723 | 131 |  |
| E_3_ | SRR11856722 | 185 |  |
| A_7_ | SRR11856721 | 197 | 137 |
| A_8_ | SRR11856720 | 124 |  |
| A_9_ | SRR11856719 | 89 |  |
| B_7_ | SRR11856718 | 340 | 315 |
| B_8_ | SRR11856717 | 167 |  |
| B_9_ | SRR11856716 | 439 |  |
| C_3_ | SRR11858530 | 210 | 335 |
| C_6_ | SRR11858539 | 430 |  |
| C_9_ | SRR11858538 | 366 |  |
| D_7_ | SRR11858537 | 229 | 233 |
| D_8_ | SRR11858536 | 103 |  |
| D_9_ | SRR11858535 | 366 |  |
| F_1_ | SRR5469191 | 82 | 79 |
| F_2_ | SRR5469170 | 143 |  |
| F_3_ | SRR5469097 | 69 |  |
| F_4_ | SRR5469171 | 66 |  |
| F_5_ | SRR5469169 | 47 |  |
| F_6_ | SRR5469154 | 67 |  |

Supplementary Table S2 The number of microorganisms at different sampling locations and sampling times in the analog cabin

| Sample serial | LB  （CFU/100 cm^2^) | R_2_A  （CFU/100 cm^2^) | PDA  （CFU/100 cm^2^) | Total count  (cells/100 cm^2^)  (BacLight^TM^) | Live count  (cells/100 cm^2^)  (BacLight^TM^) | Living cells  Proportion |
| --- | --- | --- | --- | --- | --- | --- |
| A_1_,A_2_,A_3_ | 55.1±92.4 | 9.2±27.54 | 146.9±107.6 | （9.17±2.48）×10^4^ | （7.40±2.11）×10^4^ | 80.70% |
| A_4_,A_5_,A_6_ | 1221.3±560.2 | 477.5±263.9 | 477.5±263.9 | （1.31±0.27）×10^5^ | （1.05±0.23）×10^5^ | 80.15% |
| A_7_,A_8_,A_9_ | 394.9±158.9 | 780.5±648.3 | 275.5±160.0 | （1.74±0.26）×10^5^ | （1.26±0.14）×10^5^ | 72.41% |
| B_1_,B_2_,B_3_ | 578.5±298.0 | 495.9±210.7 | 431.6±232.9 | （8.67±1.31）×10^4^ | （7.35±1.13）×10^4^ | 84.78% |
| B_4_,B_5_,B_6_ | 3057.9±1746.3 | 3810.8±2210.0 | 312.2±174.2 | （6.69±1.51）×10^4^ | （5.42±1.13）×10^4^ | 81.02% |
| B_7_,B_8_,B_9_ | 12910.9±8482.9 | 13507.8±9704.2 | 422.4±208.4 | （1.33±0.19）×10^5^ | （1.05±0.15）×10^5^ | 78.95% |
| C_1_,C_4_,C_7_ | 1037.6±465.9 | 1120.3±390.1 | 385.7±116.9 | （8.77±1.38）×10^4^ | （6.69±1.12）×10^4^ | 76.28% |
| C_2_,C_5_,C_8_ | 5417.8±5297.8 | 3278.2±1847.5 | 257.1±120.1 | （7.25±3.31）×10^4^ | （5.12±1.96）×10^4^ | 70.62% |
| C_3_,C_6_,C_9_ | 1662.1±377.5 | 1634.5±884.1 | 73.5±49.7 | （1.59±0.20）×10^5^ | （1.22±0.13）×10^5^ | 76.73% |
| D_1_,D_2_,D_3_ | 2249.8±467.1 | 1809.0±379.7 | 156.1±139.8 | （8.26±1.26）×10^4^ | （6.49±0.85）×10^4^ | 78.57% |
| D_4_,D_5_,D_6_ | 2295.7±835.5 | 1873.3±594.5 | 266.3±107.6 | （8.97±4.05）×10^4^ | （6.29±2.30）×10^4^ | 70.12% |
| D_7_,D_8_,D_9_ | 2497.7±486.8 | 2102.8±458.5 | 1000.9±917.0 | （1.38±0.42）×10^5^ | （1.04±0.33）×10^5^ | 75.36% |
| E_1_,E_2_,E_3_ | 101.0±68.9 | 64.4±90.3 | 3921.0±1611.6 | （4.21±2.28）×10^4^ | （3.09±1.72）×10^4^ | 73.40% |

Supplementary Table S3 ANOSIM analysis between groups using the Bray-Curtis distances

| Groups | *R* | *P* |
| --- | --- | --- |
| B-A | -0.0370 | 0.717 |
| B-C | -0.0370 | 0.786 |
| B-D | 0.1481 | 0.313 |
| B-E | -0.0741 | 0.698 |
| B-F | 1.0000 | 0.016 |
| A-C | 0.0370 | 0.619 |
| A-D | 0.4815 | 0.101 |
| A-E | -0.0370 | 0.600 |
| A-F | 1.0000 | 0.019 |
| C-D | 0.1111 | 0.397 |
| C-E | -0.1481 | 0.621 |
| C-F | 1.0000 | 0.009 |
| D-E | -0.0370 | 0.617 |
| D-F | 1.0000 | 0.011 |
| E-F | 1.0000 | 0.016 |

Supplementary Table S4 Network *p* value

| ASV 1 | ASV 2 | ASV 4 | ASV 3 | ASV 7 | ASV 8 | ASV 5 | ASV 9 | ASV 12 | ASV 6 | ASV 14 | ASV 16 | ASV 10 | ASV 11 | ASV 13 | ASV 15 | ASV 20 | ASV 21 | ASV 17 | ASV 19 |  |
| --- | --- | --- | --- | --- | --- | --- | --- | --- | --- | --- | --- | --- | --- | --- | --- | --- | --- | --- | --- | --- |
| ASV 1 | NA | 0 | 0 | 0 | 0 | 0 | 0 | 0 | 0 | 0 | 0 | 0 | 0 | 0 | 0 | 0 | 0 | 0 | 0 | 0 |
| ASV 2 | 0.00016512 | NA | 0 | 0 | 0 | 0 | 0 | 0 | 0 | 0 | 0 | 0 | 0 | 0 | 0 | 0 | 0 | 0 | 0 | 0 |
| ASV 4 | 3.28E-05 | 0.001137 | NA | 0 | 0 | 0 | 0 | 0 | 0 | 0 | 0 | 0 | 0 | 0 | 0 | 0 | 0 | 0 | 0 | 0 |
| ASV 3 | 0.00917926 | 0.165161 | 0.113039 | NA | 0 | 0 | 0 | 0 | 0 | 0 | 0 | 0 | 0 | 0 | 0 | 0 | 0 | 0 | 0 | 0 |
| ASV 7 | 0.00016512 | 0.000685 | 0.00297 | 0.484384 | NA | 0 | 0 | 0 | 0 | 0 | 0 | 0 | 0 | 0 | 0 | 0 | 0 | 0 | 0 | 0 |
| ASV 8 | 3.28E-05 | 0.000652 | 0.005306 | 0.456765 | 5.55E-13 | NA | 0 | 0 | 0 | 0 | 0 | 0 | 0 | 0 | 0 | 0 | 0 | 0 | 0 | 0 |
| ASV 5 | 8.79E-13 | 0.000165 | 3.28E-05 | 0.009179 | 0.000165 | 3.28E-05 | NA | 0 | 0 | 0 | 0 | 0 | 0 | 0 | 0 | 0 | 0 | 0 | 0 | 0 |
| ASV 9 | 0.00016512 | 0.000433 | 0.000369 | 0.027604 | 0.010712 | 0.008957 | 0.000165 | NA | 0 | 0 | 0 | 0 | 0 | 0 | 0 | 0 | 0 | 0 | 0 | 0 |
| ASV 12 | 0.00016512 | 0.00918 | 0.000131 | 0.09865 | 0.002052 | 0.002738 | 0.000165 | 6.69E-05 | NA | 0 | 0 | 0 | 0 | 0 | 0 | 0 | 0 | 0 | 0 | 0 |
| ASV 6 | 2.60E-14 | 0.000165 | 3.28E-05 | 0.009179 | 0.000165 | 3.28E-05 | 1.73E-11 | 0.000165 | 0.000165 | NA | 0 | 0 | 0 | 0 | 0 | 0 | 0 | 0 | 0 | 0 |
| ASV 14 | 0.00016512 | 0.000402 | 0.000428 | 0.030923 | 0.019816 | 0.027002 | 0.000165 | 3.34E-05 | 1.75E-05 | 0.000165 | NA | 0 | 0 | 0 | 0 | 0 | 0 | 0 | 0 | 0 |
| ASV 16 | 3.28E-05 | 0.002738 | 0.001209 | 0.000652 | 0.011882 | 0.010995 | 3.28E-05 | 1.07E-08 | 3.78E-06 | 3.28E-05 | 1.76E-05 | NA | 0 | 0 | 0 | 0 | 0 | 0 | 0 | 0 |
| ASV 10 | 0 | 0.000165 | 3.28E-05 | 0.009179 | 0.000165 | 3.28E-05 | 2.65E-12 | 0.000165 | 0.000165 | 1.07E-14 | 0.000165 | 3.28E-05 | NA | 0 | 0 | 0 | 0 | 0 | 0 | 0 |
| ASV 11 | 1.73E-11 | 0.000165 | 3.28E-05 | 0.009179 | 0.000165 | 3.28E-05 | 4.00E-15 | 0.000165 | 0.000165 | 1.25E-13 | 0.000165 | 3.28E-05 | 4.39E-12 | NA | 0 | 0 | 0 | 0 | 0 | 0 |
| ASV 13 | 3.08E-10 | 0.000165 | 3.28E-05 | 0.009179 | 0.000165 | 3.28E-05 | 2.65E-12 | 0.000165 | 0.000165 | 7.09E-12 | 0.000165 | 3.28E-05 | 1.63E-10 | 2.60E-14 | NA | 0 | 0 | 0 | 0 | 0 |
| ASV 15 | 1.33E-14 | 0.000163 | 3.23E-05 | 0.009136 | 0.000163 | 3.23E-05 | 4.86E-14 | 0.000163 | 0.000163 | 5.11E-15 | 0.000163 | 3.23E-05 | 4.12E-13 | 6.34E-12 | 2.90E-11 | NA | 0 | 0 | 0 | 0 |
| ASV 20 | 0.00016512 | 0.001395 | 0.000369 | 0.075699 | 0.006045 | 0.006969 | 0.000165 | 2.82E-10 | 1.75E-05 | 0.000165 | 1.24E-05 | 2.75E-07 | 0.000165 | 0.000165 | 0.000165 | 0.000163 | NA | 0 | 0 | 0 |
| ASV 21 | 0.00016386 | 0.001845 | 5.81E-08 | 0.048595 | 0.00048 | 0.002182 | 0.000164 | 0.004768 | 0.000497 | 0.000164 | 0.011895 | 0.002091 | 0.000164 | 0.000164 | 0.000164 | 0.000162 | 0.002457 | NA | 0 | 0 |
| ASV 17 | 7.09E-12 | 0.000165 | 3.28E-05 | 0.009179 | 0.000165 | 3.28E-05 | 0 | 0.000165 | 0.000165 | 3.08E-10 | 0.000165 | 3.28E-05 | 2.61E-11 | 8.79E-13 | 2.65E-12 | 5.63E-13 | 0.000165 | 0.000164 | NA | 0 |
| ASV 19 | 0.0177929 | 0.511434 | 0.006899 | 0.029292 | 0.072771 | 0.09336 | 0.017793 | 0.000891 | 0.000453 | 0.017793 | 0.020362 | 0.000116 | 0.017793 | 0.017793 | 0.017793 | 0.017725 | 0.003896 | 0.01358 | 0.017793 | NA |

| ASV 1 | ASV 2 | ASV 4 | ASV 3 | ASV 7 | ASV 8 | ASV 5 | ASV 9 | ASV 12 | ASV 6 | ASV 14 | ASV 16 | ASV 10 | ASV 11 | ASV 13 | ASV 15 | ASV 20 | ASV 21 | ASV 17 | ASV 19 |  |
| --- | --- | --- | --- | --- | --- | --- | --- | --- | --- | --- | --- | --- | --- | --- | --- | --- | --- | --- | --- | --- |
| ASV 1 | NA | 0 | 0 | 0 | 0 | 0 | 0 | 0 | 0 | 0 | 0 | 0 | 0 | 0 | 0 | 0 | 0 | 0 | 0 | 0 |
| ASV 2 | 0.00016512 | NA | 0 | 0 | 0 | 0 | 0 | 0 | 0 | 0 | 0 | 0 | 0 | 0 | 0 | 0 | 0 | 0 | 0 | 0 |
| ASV 4 | 3.28E-05 | 0.001137 | NA | 0 | 0 | 0 | 0 | 0 | 0 | 0 | 0 | 0 | 0 | 0 | 0 | 0 | 0 | 0 | 0 | 0 |
| ASV 3 | 0.00917926 | 0.165161 | 0.113039 | NA | 0 | 0 | 0 | 0 | 0 | 0 | 0 | 0 | 0 | 0 | 0 | 0 | 0 | 0 | 0 | 0 |
| ASV 7 | 0.00016512 | 0.000685 | 0.00297 | 0.484384 | NA | 0 | 0 | 0 | 0 | 0 | 0 | 0 | 0 | 0 | 0 | 0 | 0 | 0 | 0 | 0 |
| ASV 8 | 3.28E-05 | 0.000652 | 0.005306 | 0.456765 | 5.55E-13 | NA | 0 | 0 | 0 | 0 | 0 | 0 | 0 | 0 | 0 | 0 | 0 | 0 | 0 | 0 |
| ASV 5 | 8.79E-13 | 0.000165 | 3.28E-05 | 0.009179 | 0.000165 | 3.28E-05 | NA | 0 | 0 | 0 | 0 | 0 | 0 | 0 | 0 | 0 | 0 | 0 | 0 | 0 |
| ASV 9 | 0.00016512 | 0.000433 | 0.000369 | 0.027604 | 0.010712 | 0.008957 | 0.000165 | NA | 0 | 0 | 0 | 0 | 0 | 0 | 0 | 0 | 0 | 0 | 0 | 0 |
| ASV 12 | 0.00016512 | 0.00918 | 0.000131 | 0.09865 | 0.002052 | 0.002738 | 0.000165 | 6.69E-05 | NA | 0 | 0 | 0 | 0 | 0 | 0 | 0 | 0 | 0 | 0 | 0 |
| ASV 6 | 2.60E-14 | 0.000165 | 3.28E-05 | 0.009179 | 0.000165 | 3.28E-05 | 1.73E-11 | 0.000165 | 0.000165 | NA | 0 | 0 | 0 | 0 | 0 | 0 | 0 | 0 | 0 | 0 |
| ASV 14 | 0.00016512 | 0.000402 | 0.000428 | 0.030923 | 0.019816 | 0.027002 | 0.000165 | 3.34E-05 | 1.75E-05 | 0.000165 | NA | 0 | 0 | 0 | 0 | 0 | 0 | 0 | 0 | 0 |
| ASV 16 | 3.28E-05 | 0.002738 | 0.001209 | 0.000652 | 0.011882 | 0.010995 | 3.28E-05 | 1.07E-08 | 3.78E-06 | 3.28E-05 | 1.76E-05 | NA | 0 | 0 | 0 | 0 | 0 | 0 | 0 | 0 |
| ASV 10 | 0 | 0.000165 | 3.28E-05 | 0.009179 | 0.000165 | 3.28E-05 | 2.65E-12 | 0.000165 | 0.000165 | 1.07E-14 | 0.000165 | 3.28E-05 | NA | 0 | 0 | 0 | 0 | 0 | 0 | 0 |
| ASV 11 | 1.73E-11 | 0.000165 | 3.28E-05 | 0.009179 | 0.000165 | 3.28E-05 | 4.00E-15 | 0.000165 | 0.000165 | 1.25E-13 | 0.000165 | 3.28E-05 | 4.39E-12 | NA | 0 | 0 | 0 | 0 | 0 | 0 |
| ASV 13 | 3.08E-10 | 0.000165 | 3.28E-05 | 0.009179 | 0.000165 | 3.28E-05 | 2.65E-12 | 0.000165 | 0.000165 | 7.09E-12 | 0.000165 | 3.28E-05 | 1.63E-10 | 2.60E-14 | NA | 0 | 0 | 0 | 0 | 0 |
| ASV 15 | 1.33E-14 | 0.000163 | 3.23E-05 | 0.009136 | 0.000163 | 3.23E-05 | 4.86E-14 | 0.000163 | 0.000163 | 5.11E-15 | 0.000163 | 3.23E-05 | 4.12E-13 | 6.34E-12 | 2.90E-11 | NA | 0 | 0 | 0 | 0 |
| ASV 20 | 0.00016512 | 0.001395 | 0.000369 | 0.075699 | 0.006045 | 0.006969 | 0.000165 | 2.82E-10 | 1.75E-05 | 0.000165 | 1.24E-05 | 2.75E-07 | 0.000165 | 0.000165 | 0.000165 | 0.000163 | NA | 0 | 0 | 0 |
| ASV 21 | 0.00016386 | 0.001845 | 5.81E-08 | 0.048595 | 0.00048 | 0.002182 | 0.000164 | 0.004768 | 0.000497 | 0.000164 | 0.011895 | 0.002091 | 0.000164 | 0.000164 | 0.000164 | 0.000162 | 0.002457 | NA | 0 | 0 |
| ASV 17 | 7.09E-12 | 0.000165 | 3.28E-05 | 0.009179 | 0.000165 | 3.28E-05 | 0 | 0.000165 | 0.000165 | 3.08E-10 | 0.000165 | 3.28E-05 | 2.61E-11 | 8.79E-13 | 2.65E-12 | 5.63E-13 | 0.000165 | 0.000164 | NA | 0 |
| ASV 19 | 0.0177929 | 0.511434 | 0.006899 | 0.029292 | 0.072771 | 0.09336 | 0.017793 | 0.000891 | 0.000453 | 0.017793 | 0.020362 | 0.000116 | 0.017793 | 0.017793 | 0.017793 | 0.017725 | 0.003896 | 0.01358 | 0.017793 | NA |

Supplementary Table S5 Network *r* value
