# Supplementary material for: Microbiome in a ground-based analog cabin of China Space Station during a 50-day human occupation
Source: ISME Commun. 2024 Jan 24;4(1):ycae013. doi: 10.1093/ismeco/ycae013 (PMC10942772; doi:10.1093/ismeco/ycae013)
Supplement: additional_file_2_ycae013 [file additional_file_2_ycae013.docx]

**ADDITIONAL FILE 2**


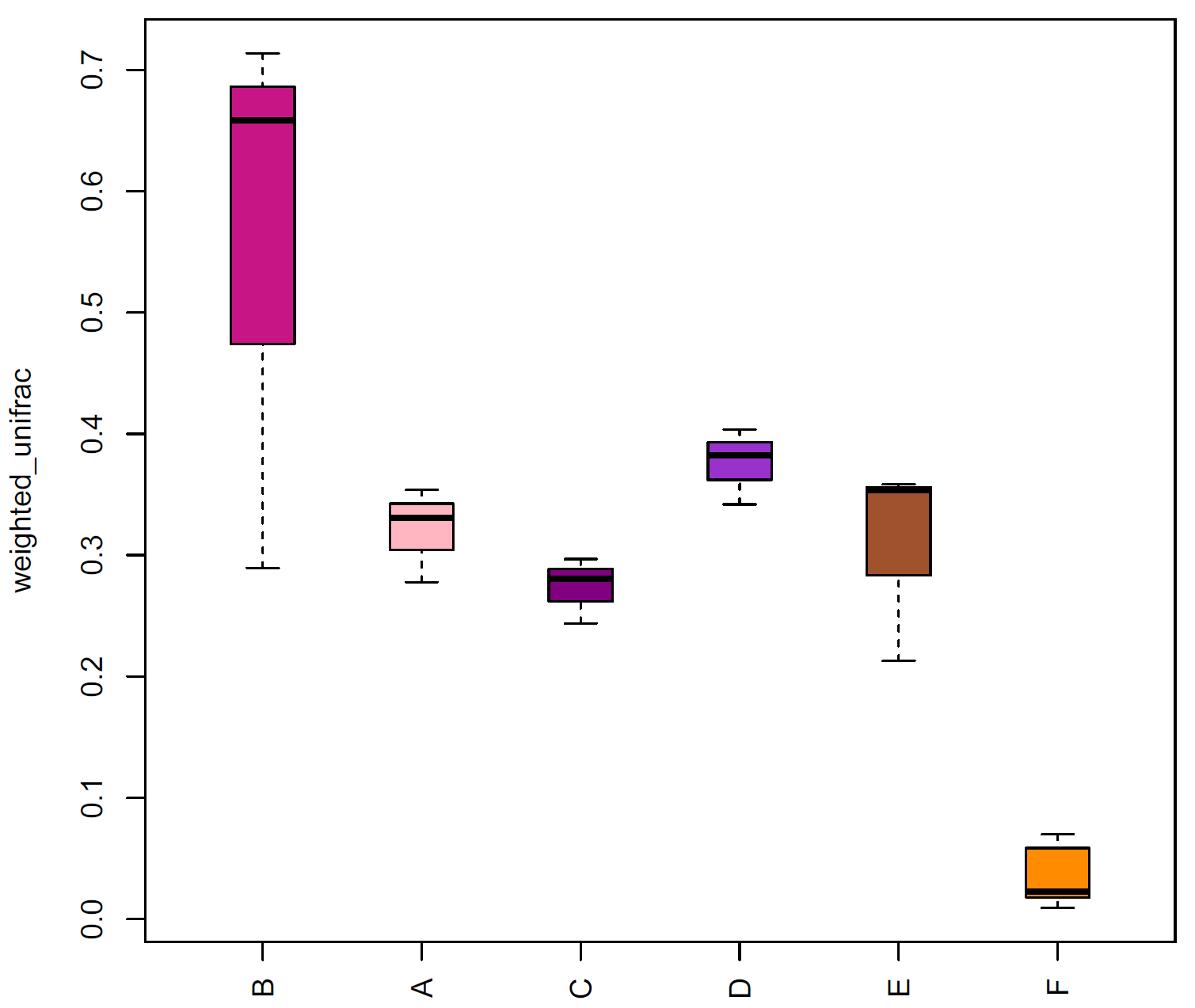


Supplementary Fig. S1 The box plot based on unifrac distance.

Supplementary Fig. S2 Rarefaction curve of 21 samples.

Supplementary Fig. S3 Shannon-Wiener curve of 21 samples.


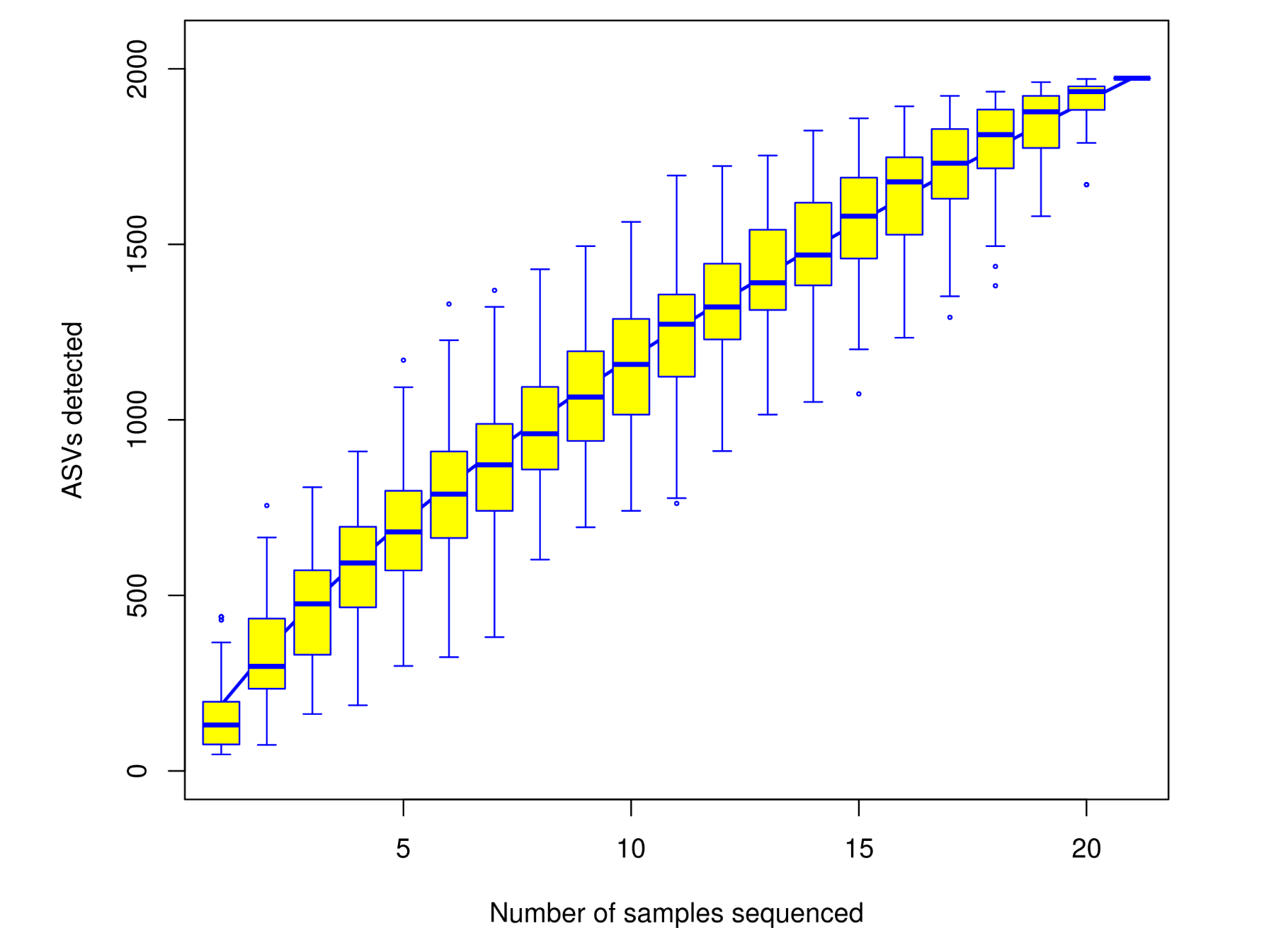


Supplementary Fig. S4 Species accumulation curves.

Supplementary Fig. S5 Rank abundance curves
